# Inertial sensor real-time feedback enhances the learning of cervical spine manipulation: a prospective study

**DOI:** 10.1186/1472-6920-14-120

**Published:** 2014-06-19

**Authors:** Antonio I Cuesta-Vargas, Jonathan Williams

**Affiliations:** 1 Departamento de Psiquiatría y Fisioterapia, Facultad de Ciencias de la Salud, Universidad de Málaga, Andalucia Tech, Cátedra de Fisioterapia y Discapacidad, Instituto de Biomedicina de Málaga (IBIMA), Grupo de Clinimetria (AE-14), Malaga, Spain; 2 School of Clinical Science, Faculty of Health, Queensland University of Technology, Brisbane, Australia; 3 School of Health and Social Care, Bournemouth University, Bournemouth, UK

**Keywords:** Medical education, Manipulation, Cervical, Kinematics, Inertial sensor

## Abstract

**Background:**

Cervical Spinal Manipulation (CSM) is considered a high-level skill of the central nervous system because it requires bimanual coordinated rhythmical movements therefore necessitating training to achieve proficiency. The objective of the present study was to investigate the effect of real-time feedback on the performance of CSM.

**Methods:**

Six postgraduate physiotherapy students attending a training workshop on Cervical Spine Manipulation Technique (CSMT) using inertial sensor derived real-time feedback participated in this study. The key variables were pre-manipulative position, angular displacement of the thrust and angular velocity of the thrust. Differences between variables before and after training were investigated using t-tests.

**Results:**

There were no significant differences after training for the pre-manipulative position (rotation p = 0.549; side bending p = 0.312) or for thrust displacement (rotation p = 0.247; side bending p = 0.314). Thrust angular velocity demonstrated a significant difference following training for rotation (pre-training mean (sd) 48.9°/s (35.1); post-training mean (sd) 96.9°/s (53.9); p = 0.027) but not for side bending (p = 0.521).

**Conclusion:**

Real-time feedback using an inertial sensor may be valuable in the development of specific manipulative skill. Future studies investigating manipulation could consider a randomized controlled trial using inertial sensor real time feedback compared to traditional training.

## Background

Spinal Manipulation can be defined as the manual application of physical impulses that are executed over a relatively short period of time (approximately 100 to 200 milliseconds) [1]. These forces and movements are designed to elicit motion within the spinal functional unit, altering the local distribution of stress through the tissues and influence pain and discomfort [1].

Cervical Spine Manipulation (CSM) is considered a high-level skill of the central nervous system because it involves bimanual coordinated rhythmical movements necessitating training to achieve proficiency [2]. Force is applied in different directions depending on the different prominences of the vertebrae to be moved [3]. An important component in learning a motor skill and improving its implementation is to detect and correct errors [4]. However, auto-detection and correction is difficult when the motor skill requirements are such that an educator cannot provide specific or immediate feedback [5]. CSM is commonly included in the curriculum of the manual therapies (Physiotherapy, Osteopathy and Chiropractic) [6]. Students are expected to practice autonomous manual therapy in clinical practice, therefore competence is essential when implementing spinal manipulation [7-11].

Despite being widely used and taught, the application of forces and hand positions during spinal manipulation varies greatly among therapists [12]. Students usually learn spinal manipulation through teacher demonstration, followed by practising the technique for themselves [13]. This is usually completed without objective feedback on key parameters such as force, velocity, time and acceleration. It has been observed that such students tend to struggle to differentiate these basic parameters of spinal manipulation [14], therefore it is necessary to search for new training methods that increase the perception and feedback of performance of spinal manipulation.

The inclusion of real time feedback on key parameters of spinal manipulation like velocity, time, acceleration and force [11] may enhance the learning process through improved perception of the parameters considered important for spinal manipulation. This in turn may improve the specificity of the technique and minimise variation [15]. Instrumentation has been utilised to provide real-time feedback for manipulation, however the methods used are often costly, environmentally constrained or utilise a mannequin [16]. The uptake of current instrumentation methods are sparse suggesting the need for a low cost, portable method, which does not interfere with the manipulation technique. One solution that has been suggested uses an inertial sensor [17-20].

No studies have been conducted to investigate the effect of using an inertial sensor as real-time feedback on the learning of CSM in physiotherapy students. The aim of the present study was to investigate the effect of real-time feedback on the performance of CSM. The null hypothesis was that training using real-time feedback will not alter kinematics employed during novice CSM.

## Methods

This study used a prospective cohort study design. Six postgraduate physiotherapy students attended a training workshop on Cervical Spine Manipulation Technique (CSMT) using inertial sensor derived real-time feedback. The specifics regarding the CSMT have been described elsewhere [19,20]. The workshop focussed on learning the cervical upslope manipulation technique, described previously [19-21]. All manipulations were targeted to the C4/5 segment and all individuals were asymptomatic. Six individuals were recruited from the University of Malaga (3 female; mean age 28.5 years; mean height 173.8 cm; mean weight 67.3 kg). All were screened for the presence of neck pain, VBI or any other contraindications for manipulation. All participants gave written informed consent and the University of Malaga ethics committee granted ethical approval.

### Instrumentation

A single inertial sensor combining tri-axial accelerometers, tri-axial rate gyroscopes and tri-axial magnetometers was used to provide kinematic measurement (Inertiacube3, InterSense Inc., MA). The sensor measured 26.2 mm × 39.2 mm × 14.8 mm and was attached so that the axes of the sensor matched the motion of the head, resulting in change in yaw representing side-bending, change in roll representing rotation and change in pitch representing flexion/extension. Data were collected at 100 Hz and stored for later processing.

### Procedure

Participants’ height and weight were recorded and in supine the sensor was attached to the forehead, positioned centrally between the eyebrows, using double sided tape. A single cervical manipulation was performed to the left and right side of the cervical spine. The order of manipulation was decided by coin toss. Only one manipulation per side was performed even in the absence of cavitation.

### Training procedures

**Figure 1:**
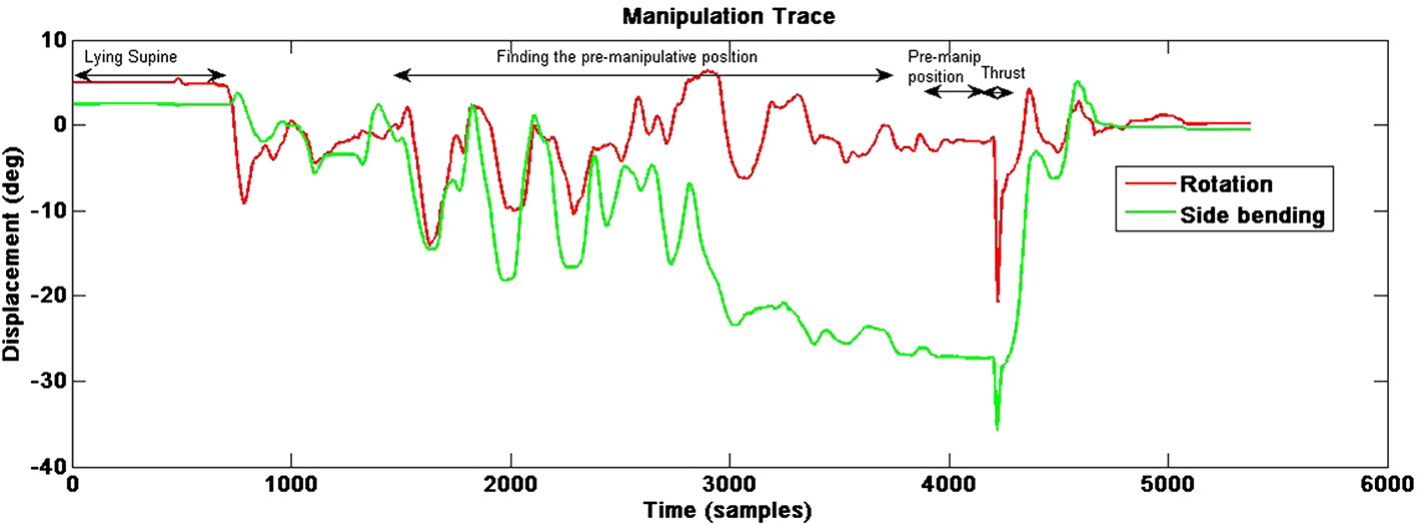
Real time feedback learning method for cervival spinal manipaulation upslope.

The training was divided into three distinct phases. All individuals performed three repetitions of CSM on their colleague, as they perceived it should be completed. In the first phase, students received an explanation and demonstration by the lecturer (physiotherapist with over 15 years experience in manual therapy). In the second phase the lecturer completed an instrumented manipulation and used the trace to identify and highlight the variables of importance for CSM. The students then practised the manipulations for 4 sessions of 60 minutes with a student-tutor ratio of 1:6. In the third phase, students received real-time feedback on their CSM execution using the graphical output from the inertial sensor. Finally, all students performed three more manipulations using the same peer. A minimum of 45 minutes was given between manipulations. A schematic of the real time feedback learning method for CSM upslope is shown in Figure 1.

### Data acquisition and processing

All raw data were transferred to Matlab (Mathworks, R2008b) for processing. Raw data were filtered using a bidirectional fourth-order, 20 Hz low–pass butterworth filter to remove high frequency noise from the signal. The change in roll and yaw from the initial position represented cervical movements of rotation and side-bending respectively with positive values assigned to the left. Pitch was not included in the analysis. The key variables were pre-manipulative position, angular displacement of the thrust and angular velocity of the thrust, which have been defined previously [19]. Differences between variables before and after training were investigated using t-tests after data were checked for normality using the Kolmogorov-Smirnov test. Statistical significance was set at p < 0.05 for all analyses.

## Results

**Figure 2:**
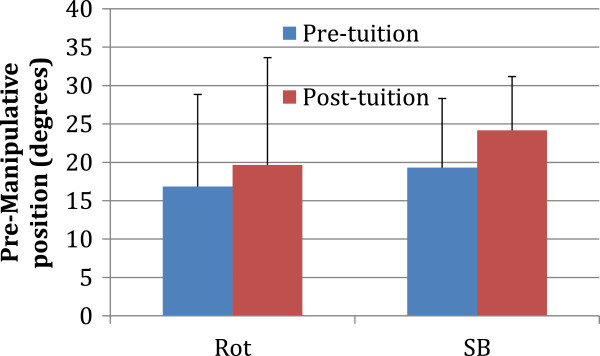
The mean and standard deviation values before (blue) and after (red) tuition are presented for pre-manipulative position (Rot, Rotation; SB, side-bending).

**Figure 3:**
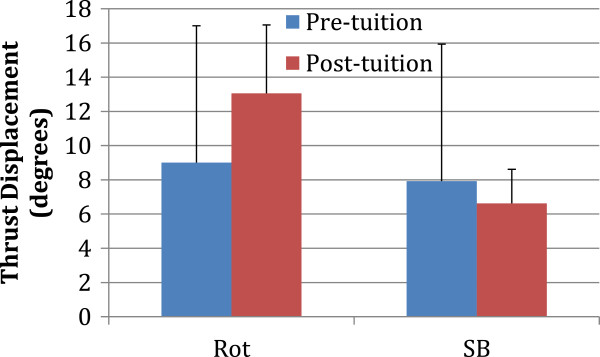
The mean and standard deviation values before (blue) and after (red) tuition are presented for angular displacement of the thrust (Rot, Rotation; SB, side-bending).

**Figure 4:**
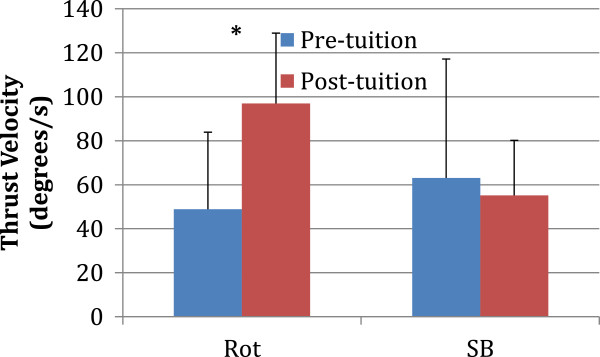
**The mean and standard deviation values before (blue) and after (red) tuition are presented for angular velocity of the thrust (Rot, Rotation; SB, side-bending).** * p<0.05.

The mean (sd) values before and after training are presented for pre-manipulative position (Figure 2), angular displacement of the thrust (Figure 3) and angular velocity of the thrust (Figure 4).

There was no significant difference after training for the pre-manipulative position regarding rotation (pre-training mean ± sd = 16.8 ± 12.4°; post-training mean ± sd = 19.6 ± 9.4; p = 0.549) or side bending (pre-training mean ± sd = 19.3 ± 13.7°); post training mean ± sd = 24.2 ± 7.4°; p = 0.312).

There was no significant difference after training for angular displacement of the thrust regarding rotation (pre-training mean ± sd = 9.0 ± 8.0°; post-training mean ± sd = 13.1 ± 8.3°; p = 0.247) or side bending (pre-training mean ± sd = 7.9 ± 3.6°; post-training mean ± sd = 6.6 ± 2.1°; p = 0.314). Thrust angular velocity demonstrated a significant difference following training for rotation (pre-training mean ± sd = 48.9 ± 35.1°/s; post-training mean ± sd = 96.9 ± 53.9°/s; p = 0.027) but not for side bending (pre-training mean ± sd = 63.1 ± 31.8°/s; post-training mean ± sd = 55.1 ± 25.3°/s; p = 0.521).

## Discussion

The objective of this study was to investigate the effect of real-time feedback on the performance of CSM. The results demonstrate that there were significant differences in the rotation angular velocity during the trust. The results also showed no significant difference in the pre-manipulative position, displacement of the thrust or side bending angular velocity.

The results obtained are consistent with previous studies that have examined the effect of real-time feedback on learning [8,14]. The improvement in velocity is in agreement with the results published by Descarreaux et al. [22], who observed that, in the context of executing a thoracic manipulation, greater velocity is associated with more experience. All students reported that using real-time feedback for learning CSM resulted in more confidence with the technique.

Training using real-time feedback seemed to result in the ability to generate greater velocity associated with the manipulative thrust. It has been suggested that velocity is a critical component to motor learning [23]. Motor learning of fast, simple movement has been studied extensively in the past [24,25]. The development of velocity has been shown to be important in the development of skill [26-28]. It has been reported that decreases in movement time and variability of movement parameters are good indicators of motor learning [23].

This study determined no increase in displacement following real-time feedback, a finding consistent with previous studies [8-11,29]. This maybe an important consideration regarding the safety of learning with such techniques as excessive displacement places the arterial structures at greater risk [30]. Real-time feedback may help to instruct the novice in minimising the resultant displacement of the spine whilst concentrating on the development of other variables which maybe important to the successful application of such techniques.

Previous studies have measured the extent of intra-subject reliability in CSM [10,18,29]. They all demonstrated high reliability in the variables observed, but there were important differences between the methods employed. One used an inertial sensor positioned on the frontal bone of a skeleton [18], while others used an instrumented treatment table [8-11,29].

Limitations of the present study include a small sample size with results for just one manipulation only, due to ethical issues of receiving multiple manipulations. It is also important to note that due to the nature of learning manipulation, a series of practise manipulations were conducted which may alter the stiffness properties of the spine being manipulated and therefore alter the kinematic profile of the technique. A mixed training method was used rather than the inertial sensor only making the interpretation of results relating specifically to the sensor feedback difficult. The current study measured global head motion not intra-segmental motion as is often the aim with manipulation. However such a system can be used to detect faults encouraging reflection and self-correction to enhance autonomous learning. Further studies can now explore the use of such a method for other regions of the spine or investigate the how manipulative skills are acquired over longer time frames.

## Conclusions

The results of this study suggest that real-time feed back derived from an inertial sensor can be used to quantity key variables associated with CSM and importantly can aid in the development of thrust velocity. Future studies investigating manipulation could consider randomized controlled trial using inertial sensors compared to traditional training.

## Competing interests

The authors declare that they have no competing interests.

## Authors’ contributions

Both authors have made contributions to conception of this study, participated in the analysis and interpretation of data and were involved in drafting the manuscript or revising it critically for important intellectual content. Both authors have given final approval of the version to be published.

## Pre-publication history

The pre-publication history for this paper can be accessed here:

http://www.biomedcentral.com/1472-6920/14/120/prepub
